# Outcomes after endovascular thrombectomy for acute ischemic stroke patients with active cancer: A systematic review and meta-analysis

**DOI:** 10.3389/fneur.2022.992825

**Published:** 2022-10-20

**Authors:** Linyan Duan, Zhaolin Fu, Hengxiao Zhao, Chengyu Song, Qiuyue Tian, Adam A. Dmytriw, Robert W. Regenhardt, Ziyi Sun, Xiaofan Guo, Xue Wang, Bin Yang

**Affiliations:** ^1^Department of Radiology and Nuclear Medicine, Xuanwu Hospital, Capital Medical University, Beijing, China; ^2^Beijing Key Laboratory of Magnetic Resonance Imaging and Brain Informatics, Beijing, China; ^3^Department of Neurosurgery, Xuanwu Hospital, Capital Medical University, Beijing, China; ^4^China International Neuroscience Institute (China-INI), Beijing, China; ^5^Department of Library, Beijing Luhe Hospital, Capital Medical University, Beijing, China; ^6^Beijing Key Laboratory of Clinical Epidemiology, School of Public Health, Capital Medical University, Beijing, China; ^7^Neuroendovascular Program, Massachusetts General Hospital and Harvard Medical School, Boston, MA, United States; ^8^Department of Neurology, Loma Linda University Health, Loma Linda, CA, United States; ^9^Department of Library, Xuanwu Hospital, Capital Medical University, Beijing, China

**Keywords:** acute ischemic stroke, endovascular thrombectomy, active cancer, meta-analysis, systematic review

## Abstract

**Background:**

Active cancer (AC) is a known risk factor for stroke and a common comorbidity among patients being considered for treatment with endovascular thrombectomy (EVT). This systematic review and meta-analysis aimed to evaluate the current evidence for the feasibility, efficacy, and safety of EVT for patients with AC.

**Methods:**

MEDLINE, EMBASE, and the Cochrane Library were searched for relevant randomized controlled trials (RCTs) and observational studies which met the inclusion criteria for EVT in patients with AC. Studies were excluded due to the mismatch of data format, article type, and group design. The risk of bias was assessed through different scales according to the study design. *I*^2^ statistics were used to evaluate the heterogeneity. Funnel plots were used to evaluate publication bias.

**Results:**

A total of six studies and 3,657 patients were included. Compared to without active cancer (WC) patients, patients with AC had a significantly higher proportion of in-hospital mortality (OR 3.24; 95% CI, 1.03–10.15). The estimated rate of favorable outcome of six studies was lower in patients with AC than in patients with WC (OR 0.47; 95% CI, 0.35–0.65). For 90-day mortality of four studies, the AC group had a higher proportion when compared with the WC group (OR 3.87; 95% CI, 2.64–5.68). There was no difference between rate of six studies of successful recanalization (OR 1.24; 95% CI, 0.90–1.72) and four studies of symptomatic ICH (OR 1.09; 95% CI, 0.61–1.97) comparing AC and WC.

**Conclusion:**

Patients with AC are less likely to have a favorable outcome and have a higher risk of mortality after EVT. Further studies are warranted for this unique patient population.

## Introduction

Cancer is a widely known risk factor of acute ischemic stroke (AIS), especially among patients with active cancer (AC) which was diagnosed within 6 months or during the admission period, requires chemotherapy or surgical treatment within 6 months, or was recurrent, metastatic, or inoperable (1). Several mechanisms related to malignancy theoretically increase the risk of AIS, such as hypercoagulation state, migratory thrombosis, and tumor embolus (2–4). Also, AC-related stroke is associated with a higher morbidity in several studies (5–8). Indeed, about 10% of hospitalized patients with AIS had AC (9–11). Unfortunately, patients with AC are often ineligible for intravenous thrombolysis (IVT) due to various reasons, such as bleeding tendency and recent prior surgery (12, 13).

Endovascular thrombectomy (EVT) has revolutionized acute stroke care and is recommended as the first-line treatment for AIS due to large vessel occlusion (LVO) (14, 15). Whether EVT benefits AC-related stroke patients to a similar degree remains uncertain. In a previous meta-analysis including a relatively limited number of studies, the AC group had a comparable rate of successful recanalization and symptomatic intracerebral hemorrhage (sICH) compared to the control group, but a lower rate of favorable outcome (modified Rankin Scale ≤ 2) and a higher rate of mortality (16). However, some recent clinical studies indicated that rate of favorable outcome may be similar between the two groups, which differs from the aforementioned meta-analysis (4, 5). Other studies suggested that patients with active cancer are more likely to have any cerebral hemorrhage (4, 6). Thus, this article aims to investigate the safety and effectiveness of EVT in AC-related stroked patients, so as to provide clinicians with the most comprehensive and updated evidence for decision-making in clinical practice.

## Methods

This study was conducted according to the statement of Preferred Reporting Items for Systematic Reviews and Meta-Analyses (PRISMA) (17).

### Search strategy

Studies for inclusion were identified by two independent reviewers (CS and ZS) from the three databases: MEDLINE, EMBASE, and the Cochrane Library. Eligible studies were restricted from database inception until 24 January 2022 in the English language. The terms “ischemic stroke”, “brain ischemia”, “cancer”, “neoplasm”, “embolectomy”, “mechanical thrombectomy”, and “endovascular thrombectomy” were applied in our search strategy for potentially relevant studies. The detailed search strategy is presented in the online Supplementary material (Supplement File 1).

### Study eligibility

The criteria for study design were specified according to the Population, Intervention, Comparison, Outcome (PICO) model.

#### Patient selection criteria

Inclusion criteria included adult patients (age ≥ 18 years) with AIS due to LVO, including anterior or posterior circulation occlusions, undergoing EVT. These were divided into active cancer (AC) group and without active cancer (WC) group according to the presence of AC. Active cancer was defined as cancer that was diagnosed within 6 months or during the admission period, requires chemotherapy or surgical treatment within 6 months, or was recurrent, metastatic, or inoperable (1). Arterial occlusion is confirmed by either computed tomographic angiography (CTA), magnetic resonance angiography (MRA), or digital subtraction angiography (DSA). We did not collect any primary data from patients, so ethics approval was deemed unnecessary by our IRB given there was a minimal patient risk.

#### Intervention

Mechanical thrombectomy with modern devices, such as stent retrievers or aspiration catheters, for patients is available to additional intravenous thrombolysis.

#### Outcomes

At least one of the following items was reported:

Primary outcomes:

1. Favorable outcome defined as modified Rankin Score (mRS) of 0–2 or equal to pre-stroke score at 90 days.
2. Symptomatic intracranial hemorrhage (SICH) was diagnosed if a new intracranial hemorrhage was associated with any of the following conditions: (1) NIHSS score increased >4 points than that immediately before worsening; (2) NIHSS score increased >2 points in one category; (3) deterioration of neurological status led to intubation, hemicraniectomy, external ventricular drain placement, or other major medical or surgical intervention, according to the second European Australasian Acute Stroke Study classification (ECASS II) (18).

Secondary outcomes:

1. Successful recanalization (MTICI 2b-3) determined by post-interventional DSA.
2. Mortality at 90-day follow-up.
3. In-hospital mortality.

#### Comparison

The comparator was patients of the WC group, who also received EVT without limitation of additional intravenous thrombolysis.

#### Studies

We included RCTs and observational studies, including cohort studies and case-controlled studies. Other types of articles such as abstracts, conference reports, and case reports were excluded. Studies which did not report the above outcomes or extractable complications were also excluded.

### Selection of studies and data extraction

Two reviewers (ZF and ZS) independently searched the databases to include eligible studies. In the initial stage of screening, titles, keywords, and abstracts were reviewed, and irrelevant studies were excluded. Subsequently, full articles of all the remaining studies were obtained and carefully reviewed to assess eligibility, and reasons for inclusion or exclusion of studies were documented in detail. Conflicts in study selection between two reviewers were resolved by a third reviewer (HZ).

The extraction of data from included studies was conducted by two independent reviewers (LD and HZ) using a standardized data extraction form. The extracted information of included studies was as follows: (1) characteristics of the study, such as publication time, country, and the number of patients; (2) demographic characteristics, such as age, gender, cancer type, medical history, laboratory results, site of occlusion by angiography, and intervention characteristics; (3) aforementioned outcomes such as sICH and favorable outcome. The resolution of disagreement regarding data extraction was achieved through the assistance of another reviewer (XB). For missing or ambiguous data in included studies, clarification of data through direct contact with the corresponding authors by e-mail was attempted.

### Assessment of risk bias and heterogeneity

Two reviewers (QT and XW) independently assessed the risk of bias for each included study. The Cochrane Collaboration criteria were applied in the process of selection of RCTs (Supplementary File 2). The Newcastle–Ottawa scale was used for observational studies, such as cohort studies and case-control studies (Supplementary File 3) (19). The heterogeneity of pooled outcomes was evaluated by *I*^2^ statistic. If *I*^2^ is <20%, the heterogeneity was considered acceptable. The Mantel–Haenszel method for fixed-effects estimation was applied if heterogeneity was mild or moderate. For substantial heterogeneity of outcomes, we conducted meta-regression and sensitivity analyses to explore the potential source of heterogeneity.

### Measures of treatment effect

A meta-analysis on a specific result was performed only when there were at least two suitable studies for analysis. If there were insufficient suitable studies for meta-analysis, the results were described in the narrative. We adapted OR with 95% CIs for dichotomous data and the mean differences (MD) with 95% CIs for continuous data. The standard of *p*-value < 0.05 was considered statistically significant. The Stata statistical software (version 15.0, Stata Corp, College Station, Texas, USA) was used for data analysis and heterogeneity assessment.

## Results

### Study selection and study characteristics

**Figure 1:**
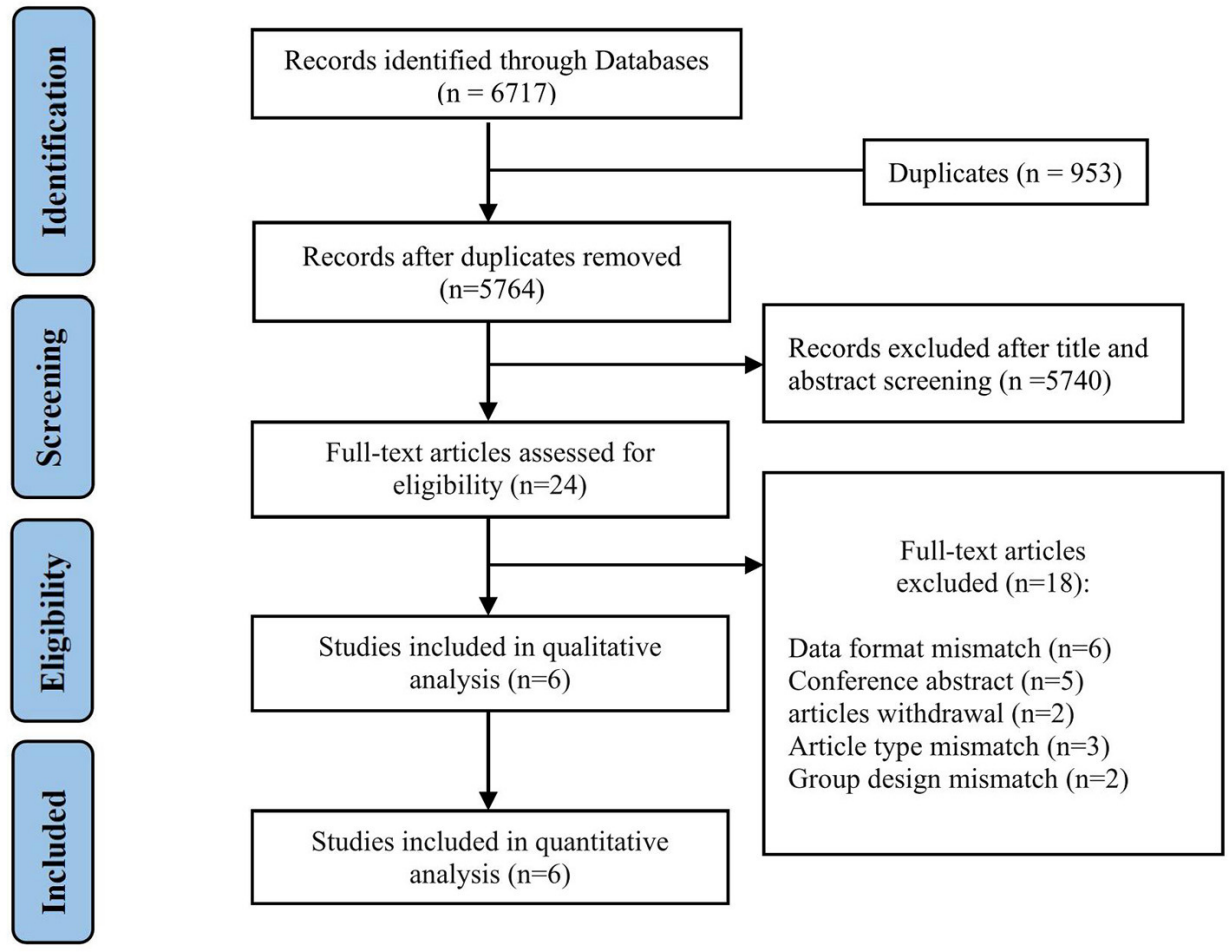
Flow diagram of literature for meta-analysis.

We found 5,764 references, abstracts, and related clinical trials from the three electronic databases and clinical trial registries. Among the results, 24 full-text articles were retrieved after initial checks, and six studies were finally eligible for inclusion in the qualitative and quantitative analysis. The process of study selection and reasons for exclusion are summarized in Figure 1. Table 1 shows the characteristics of included studies and patients. A total of six studies and 3,657 patients met inclusion criteria (3–6, 20). All studies were published after 2019, two were conducted in Italy, two were conducted in the USA, and two were performed in South Korea. There was one multicenter study, and the remaining were single-center studies. The inclusion and exclusion criteria for patients included in the individual studies are summarized in Table 2. The inclusion criteria consisted of the time window, cancer diagnosis, and baseline neurological and imaging evaluations. The number of patients in each included study ranged from 48 to 2,583, and the ratio of men to women was equal. The site of occlusion as determined by angiography was mostly located at anterior circulation, especially the internal carotid artery and M1 segment of the middle cerebral artery. The estimated times of onset to groin puncture and onset to revascularization ranged from 153–494 to 203–615 min, respectively.

**Table 1:** Baseline characteristics.

	**Bang-Hoon Cho et al. (** **3** **)**		**Verschoof et al. (** **20** **)**		**Fabrizio Sallustio et al. (** **21** **)**		**Lee et al. (** **6** **)**		**Joshi et al. (** **4** **)**		**Ciolli et al. (** **5** **)**	
**Basic information**												
Publication time	2019		2022		2019		2019		2021		2021	
Country	South Korea		United States		Italy		South Korea		United States		Italy	
Type of studies	Single center		Muticenter		Single center		Single center		Single center		Single center	
NOS score												
**Demographic characteristics**												
Number	AC (*n* = 27)	WC (*n* = 351)	AC (*n* = 124)	WC (*n* = 2459)	AC (*n* = 24)	WC (*n* = 24)	AC (*n* = 26)	WC (*n* = 227)	AC (*n* = 19)	WC (*n* = 95)	AC (*n* = 14)	WC (*n* = 267)
Gender, male, n (%)	20 (74.1)	185 (52.7)	58 (46.8)	1277 (51.9)	8 (33.3)	8 (33.3)	18 (69.2)	137 (60.4)	2 (10.5)	10 (10.5)	8 (57)	135 (51)
Age (years) mean ± SD median [IQR]	69.04 ± 9.95	70.12 ± 11.46	69 ± 11	70 ± 14	69 ± 10.1	70.7 ± 9.3	63.2 ± 11.6	68.8 ± 11.3	70.9 (11.16)	70.7 (11.4)	73 (61–78)	72 (60–79)
Baseline NIHSS mean ± SD median [IQR]	11 (7–14)	12 (9–15)	16 (12–19)	16 (11–19)	14.2 ± 5.2	14.1 ± 4.9	14 (10–18)	13 (9–17)	22 (7.5)	22 (9.5)	20 (10–23)	16 (10–21)
Baseline ASPECTS mean ± SD median [IQR]	8 (6–8.25)	8 (6–9)	9 (8–10)	9 (7–10)	9.1 ± 0.9	8.8 ± 1	NR	NR	NR	NR	NR	NR
**Cancer type**												
Digestive tract, *n* (%)	7 (25.9)	0	41 (33.1)	0	5 (20.8)	0	NR	NR	2 (10.5)	0	NR	NR
Hepatobiliary, *n* (%)	7 (25.9)	0	0	0	0	0	NR	NR	1 (5.3)	0	NR	NR
Lung, *n* (%)	6 (22.2)	0	31 (25.0)	0	8 (33.3)	0	NR	NR	6 (31.6)	0	NR	NR
Urogenital, *n* (%)	3 (11.1)	0	26 (21.0)	0	4 (16.6)	0	NR	NR	5 (26.3)	0	NR	NR
Breast, *n* (%)	1 (3.7)	0	16 (12.9)	0	4 (16.6)	0	NR	NR	3 (15.8)	0	NR	NR
Hematological, *n* (%)	3 (11.1)	0	3 (2.4)	0	1 (4.1)	0	NR	NR	2 (10.5)	0	NR	NR
Other^a^, *n* (%)	0	0	7 (5.6)	0	2 (8.2)	0	NR	NR	0	0	NR	NR
**Medical history**												
Hypertension, *n* (%)	14 (51.9)	208 (59.3)	50 (41.3)	1262 (52.3)	15 (62.5)	17 (70.8)	14 (53.8)	147 (64.8)	NR	NR	9 (64)	179 (72)
Admission SBP, mmHg mean ± SD median [IQR]	130.37 ± 21.75	137.35 ± 22.84	145 ± 25	150 ± 25	135.1 ± 22.2	145 ± 19.4	NR	NR	NR	NR	143 (135–173)	151 (138–170)
Admission DBP, mmHg mean ± SD median [IQR]	81.11 ± 12.51	85.56 ± 14.88	80 ± 16	82 ± 16	78.2 ± 13	81.1 ± 13.6	NR	NR	NR	NR	86 (76–100)	80 (70–90)
Diabetes mellitus, *n* (%)	7 (25.9)	73 (20.8)	25 (20.5)	391 (16)	8 (33.3)	5 (20.8)	7 (26.9)	56 (24.7)	NR	NR	3 (23)	51 (21)
Current smoking, *n* (%)	3 (11.1)	50 (14.2)	36 (29.8)	531 (21.8)	8 (33.3)	6 (25)	NR	NR	NR	NR	NR	NR
Dyslipidemia, *n* (%)	2 (7.4)	30 (8.5)	32 (26.7)	696 (29.5)	NR	NR	3 (11.5)	61 (26.9)	NR	NR	3 (23)	115 (48)
Atrial fibrillation, *n* (%)	6 (22.2)	117 (33.3)	32 (26.2)	582 (23.9)	7 (29.1)	9 (37.5)	6 (23.1)	118 (52.0)	NR	NR	NR	NR
Previous stroke, *n* (%)	3 (11.1)	60 (17.1)	17 (13.9)	408 (16.7)	NR	NR	3 (11.5)	47 (20.7)	NR	NR	NR	NR
**Laboratory results**												
Glucose, mean ± SD median [IQR]	NR	NR	7.4 ± 2.5	7.5 ± 2.3	142.9 ± 58.1	131.3 ± 57.8	NR	NR	NR	NR	NR	NR
Thrombocyte count, mean ± SD median [IQR]	NR	NR	272 ± 120	249 ± 83	NR	NR	212.35 ± 134.25	218.37 ± 63.52	NR	NR	NR	NR
**Loction of the occlusion**												
MCA, *n* (%)	NR	NR	NR	NR	14 (58.3)	15 (62,5)	14 (53.7)	123 (54.2)	14 (73.7)	68 (71.5)	NR	NR
M1, *n* (%)	16 (59.3)	172 (49.0)	73 (62.4)	1350 (57.5)	7 (29.2)	8 (33.3)	12 (46.2)	105 (46.3)	NR	NR	NR	NR
M2, *n* (%)	2 (7.4)	55 (15.7)	12 (10.3)	364 (15.5)	NR	NR	2 (7.7)	19 (8.4)	NR	NR	NR	NR
ICA, *n* (%)	9 (33.3)	124 (35.3)	31 (26.5)	617 (26.3)	NR	NR	15 (57.7)	67 (29.5)	5 (26.3)	19 (20)	NR	NR
Other, *n* (%)	NR	NR	1 (0.9)	17 (0.7)	NR	NR	2 (7.7)	59 (26.0)	NR	NR	NR	NR
**Intervention characteristics**												
IV thrombolysis, *n* (%)	17 (63)	203 (57.8)	69 (56.6)	1862 (75.8)	11 (45.8)	11 (45.8)	5 (19.2)	79 (35.0)	NR	8 (42)	5 (36)	127 (48)
Onset to puncture, Min mean ± SD median [IQR]	NR	NR	203 (155–258)	200 (153–260)	NR	NR	NR	NR	NR	NR	205 (168–368)	268 (195–494)
Onset to recanalization, Min mean ± SD median [IQR]	351.81 ± 170.59	341.05 ± 170.35	255 (203–335)	256 (204–320)	NR	NR	NR	NR	NR	NR	335 (260–515)	380 (290–615)

a Metastases from unknown primary tumor, malignant tumor lower leg (histopathological findings not reported), sarcoma central pulmonary artery, melanoma, Non-Hodgkin lymphoma and pancreas.

AC, active cancer; WC, without cancer; AC is defined as being diagnosed with tumor and receiving or refusing relevant therapy.

SD, standard deviation; IQR, interquartile range; IV, intravenous; ICA, intracranial carotid artery; MCA, middle cerebral artery; M1, segment of the MCA; M2, segment of the MCA; NR, not report; NIHSS, national institutes of health stroke scale; ASPECTS, Alberta stroke program early CT score.

**Table 2:** Inclusion and exclusion criteria of patients in included studies.

**Study**	**Verschoof et al.**	**Lee et al.**	**Cho et al.**	**Sallustio et al.**	**Joshi et al.**	**Ciolli et al.**
Time window	ND	Presentation within 24 h of stroke onset	Presentation within 8 h of stroke onset	ND	ND	ND
Active cancer	Cancer diagnosis within 12 months prior to stroke, metastatic disease, or cancer treatment in the last 30 days	Patients with any metastatic disease, were undergoing current treatment for a malignancy offered treatment for a malignancy, but declined	Current or previous metastatic disease; patients undergoing current treatment for malignancy; patients refused treatment for current cancer; initial diagnosis of malignancy was made during hospitalization after the onset of stroke	ND	Patients who were diagnosed with cancer and were either receiving treatment or were treated conservatively, or those who were diagnosed with but refused treatment for cancer	Cancer was considered active if the diagnosis had occurred within six months before stroke, if patients had been treated for cancer within the previous six months, or in the presence of recurrent or metastatic cancer.
Baseline neurologic evaluation	ND	ND	mRS ≤ 2	ND	ND	ND
Imaging evaluation	ND	On-enhanced cranial CT scan and multimodal MRI; DWI–perfusion-weighted imaging mismatch	No evidence of ICH on CT or MRI; major arterial occlusion on MRA or CTA; a target mismatch pattern on multimodal MRI according to visual estimation; infarct volume of less than one-third of the MCA territory on DWI or non-enhanced CT	CTA for assessment of collaterals; CT quantified by ASPECTS	A non-contrast head CT and CTA of the head and neck with or without perfusion.	CT; CTA; CTP
Exclusion criteria	Patients with a history of cancer but not fulfilling the definition of active cancer.	AIS without occlusion of the relevant artery; arterial reperfusion performed > 24 h after symptom onset; intracranial neoplasm or metastasis; other causes of AIS; failure of IAT due to technical reasons and if clinical follow-up with a modified Rankin scale (mRS) value at 90 days was unavailable	ND	ND	ND	Patients were excluded if endovascular treatment, oncologic or follow-up data were missing

ND, no documentation; CT, computed tomography; MRI, magnetic resonance imaging; DWI, Diffusion Weighted Imaging; CTA, computed tomography angiography; mRS, modified Rankin Scale; MRA, MR angiography; ASPECTS, Alberta stroke program early CT score; NIHSS, national institute of health stroke scale; CTP, CT perfusion; ICH, intracranial hemorrhage; FLAIR, fluid attenuated inversion recovery.

### Meta-analysis of primary and secondary outcomes

Table 3 shows the primary and secondary outcomes for patients with AC and WC. In four studies and 3,140 patients, compared with patients with WC, patients with AC had a significantly higher proportion of in-hospital mortality (OR, 3.24; 95% CI, 1.03–10.15, *p* = 0.030; *I*^2^ = 66.6%). The estimated rate of favorable outcome of six studies and 3,657 patients was lower in patients with AC than in patients with WC (OR, 0.47; 95% CI, 0.35–0.65, *p* = 0.547; *I*^2^ = 0.0%). The outcome of four studies and 3,345 patients of 90-day mortality showed that the AC group had a higher proportion when compared with the WC group (OR, 3.87; 95% CI, 2.64–5.68; *p* = 0.287; *I*^2^ = 19.3%). There was no difference between rate of successful recanalization (OR, 1.24; 95% CI, 0.90–1.72; *p* = 0.828; *I*^2^ = 0.0%) in six studies and 3,657 patients and symptomatic ICH (OR, 1.09; 95% CI, 0.61–1.97; *p* = 0.386; *I*^2^ = 0.0%) in four studies and 3,140 patients between groups AC and WC.

**Table 3:** The outcomes of comparison between AC and WC of primary and safety outcomes.

**Outcomes**	**OR (95% CI)**	***I^2^***	***P*** **-value**
Favorable outcome at 90-day	0.47 (0.35, 0.65)	0.0%	0.547
Rate of successful recanalization	1.24 (0.90, 1.72)	0.0%	0.828
Symptomatic ICH	1.09 (0.61, 1.97)	0.0%	0.386
90-day mortality	3.87 (2.64, 5.68)	19.3%	0.287
In-hospital mortality	3.24 (1.03, 10.15)	66.6%	0.030

CI, confidence interval; I2, the variation attributable to heterogeneity; mTICI, modified Thrombolysis in Cerebral Infarction; mRS, modified Rankin Score; ICH, Intracerebral hemorrhage.

### Risk of bias

**Figure 2:**
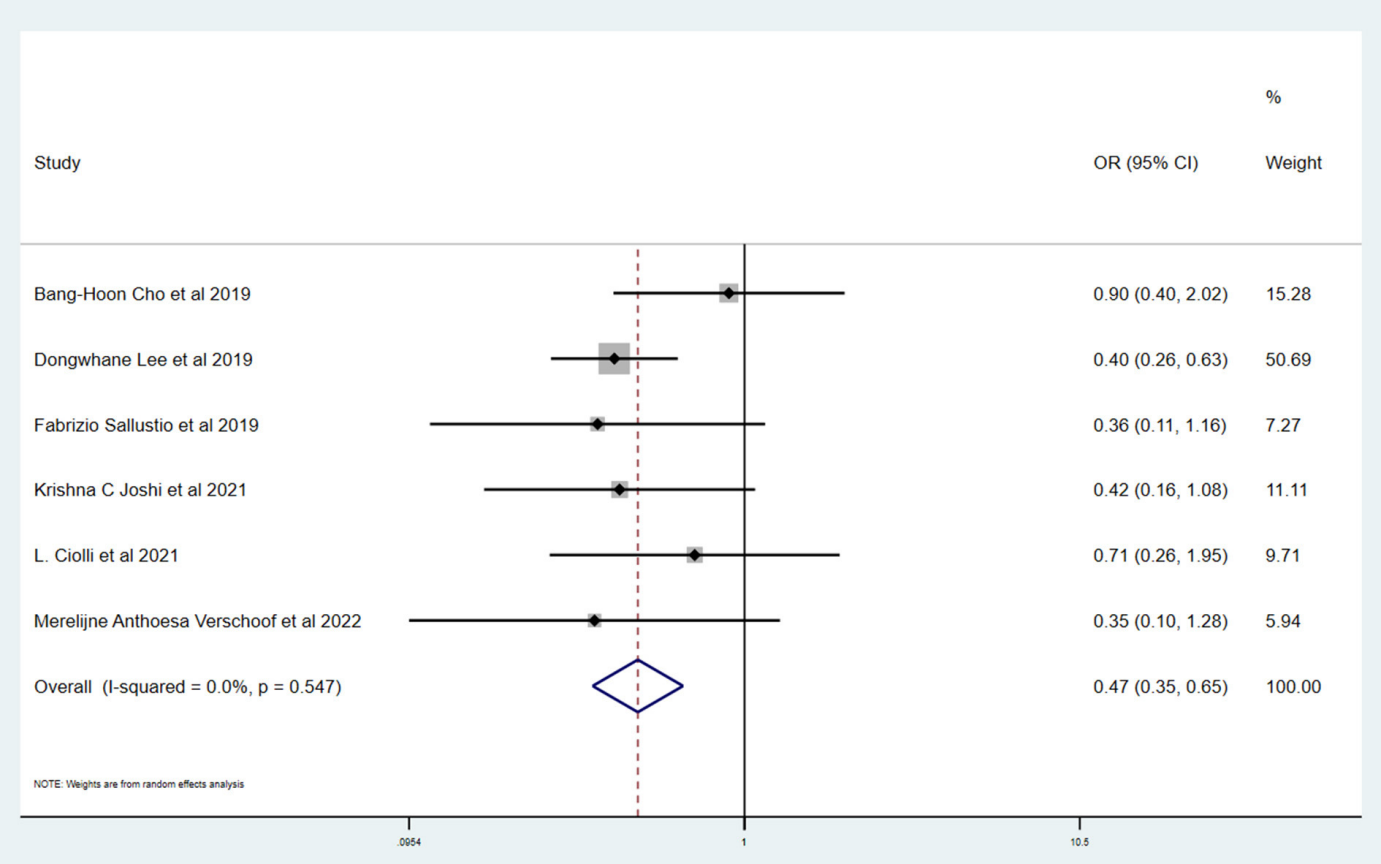
Forest plots of meta-analyses of favorable outcome at 90 days.

**Figure 3:**
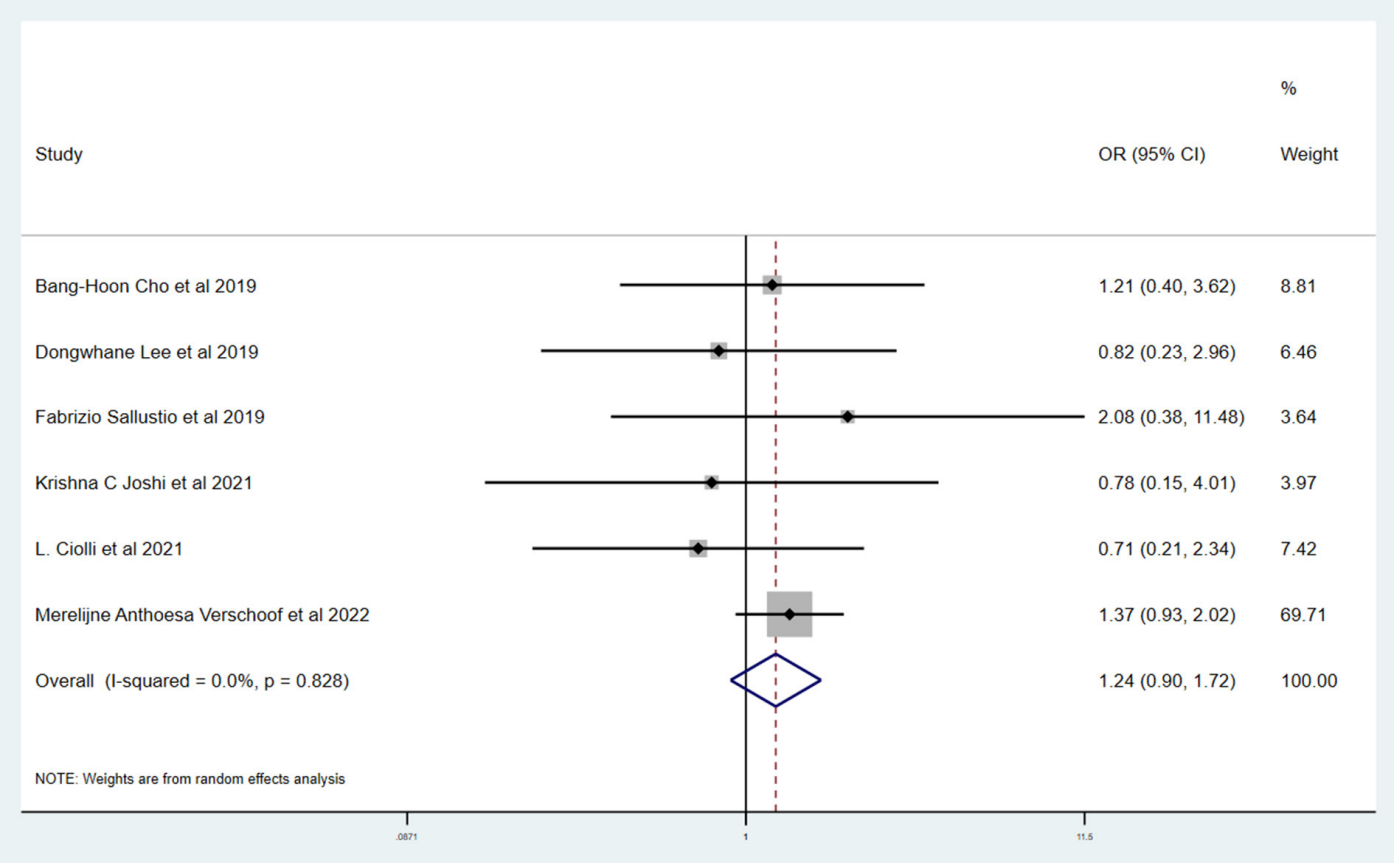
Forest plots of meta-analyses of rate of successful recanalization.

**Figure 4:**
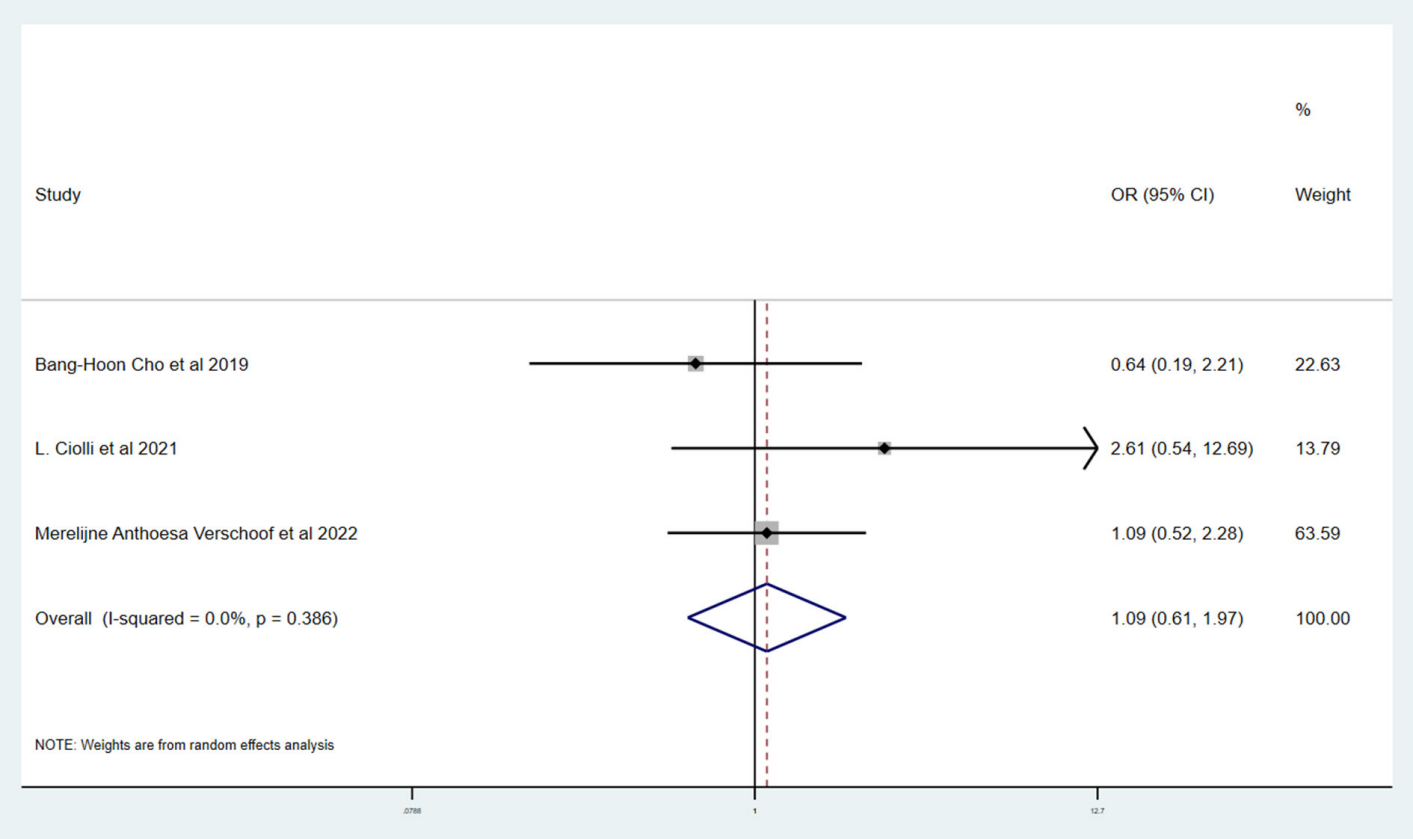
Forest plots of meta-analyses of rate of symptomatic ICH.

**Figure 5:**
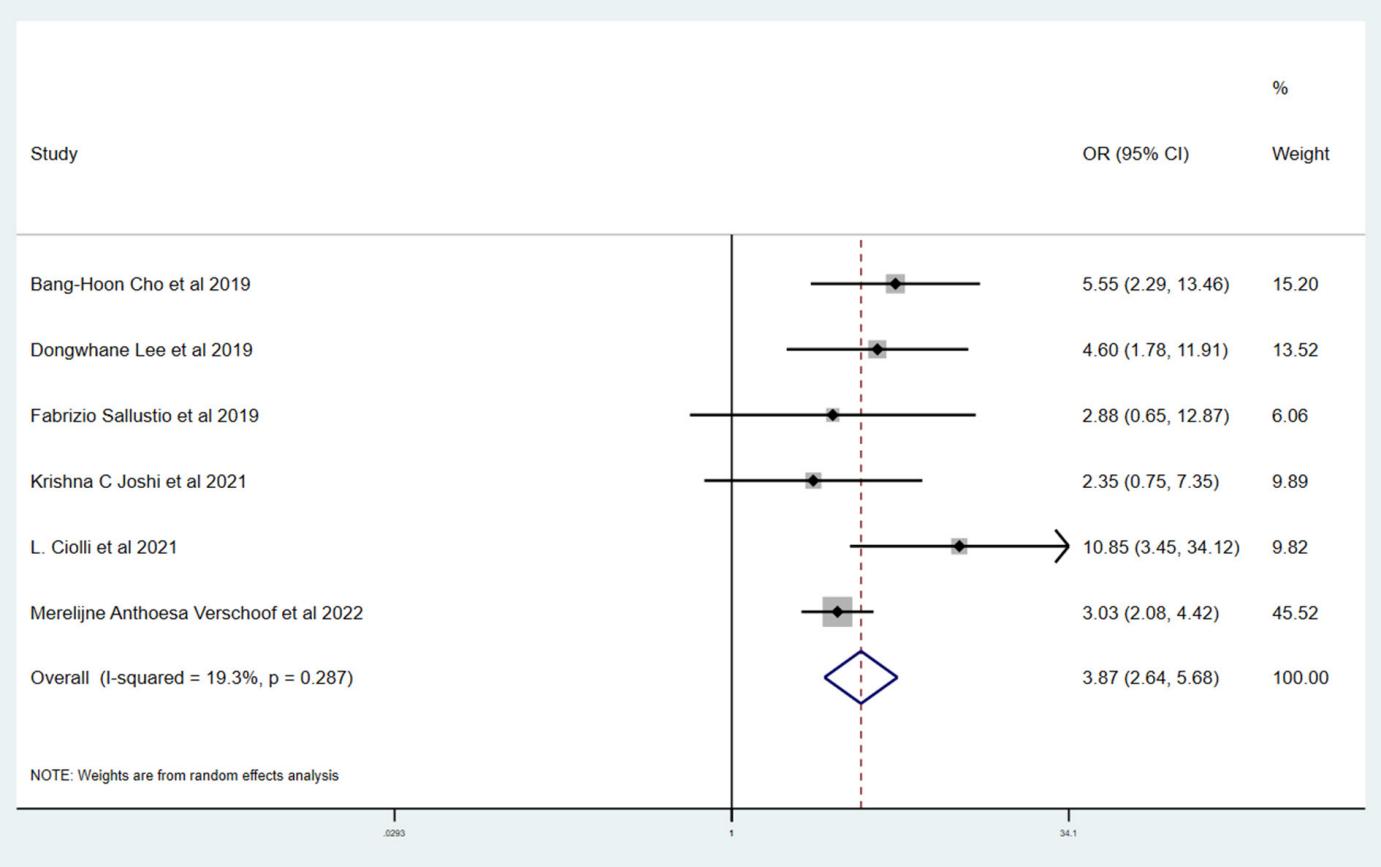
Forest plots of meta-analyses of rate of 90-day mortality.

**Figure 6:**
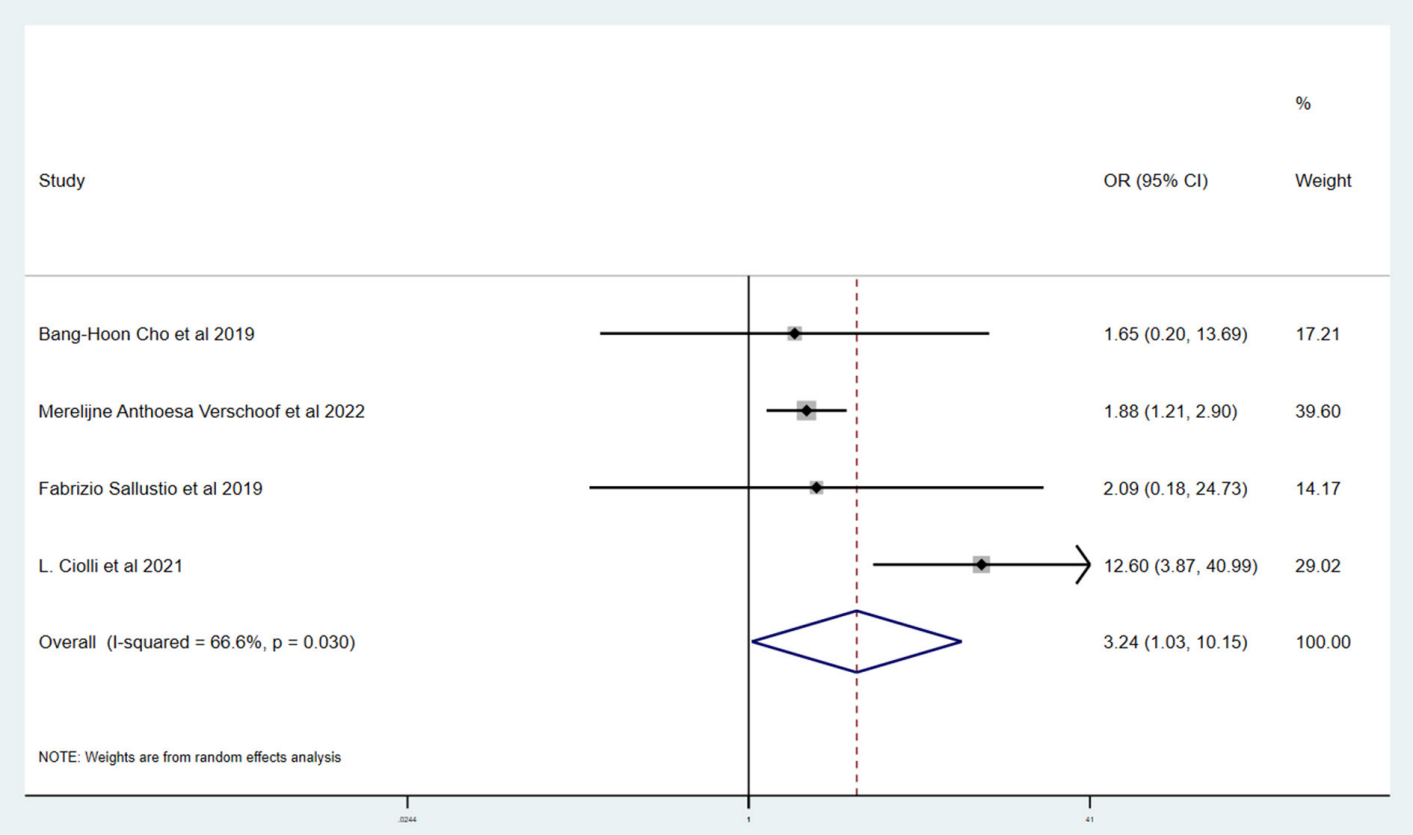
Forest plots of meta-analyses of rate of in-hospital mortality.

The Newcastle–Ottawa scale was used to assess the bias risk of observational studies, such as case-control studies, with the majority of included studies being low-risk. Funnel plots were used to explore the publication bias, with the results demonstrating no evident reporting bias. The outcomes of the above analyses are presented in Figures 2–6.

## Discussion

This systematic review and meta-analysis summarized the safety and efficacy of EVT for AIS patients with AC. The successful recanalization proportion (OR, 1.24; 95% CI, 0.90–1.72; *p* = 0.828; *I*^2^ = 0.0%) and rate of symptomatic ICH (OR, 1.09; 95% CI, 0.61–1.97; *p* = 0.386; *I*^2^ = 0.0%) in patients with AC were comparable with patients with WC. However, patients with AC had a significantly lower rate of favorable outcome (OR, 0.47; 95% CI, 0.35–0.65, *p* = 0.547; *I*^2^ = 0.0%). We also showed a tendency for a higher proportion of in-hospital mortality (OR, 3.24; 95% CI, 1.03–10.15, *p* = 0.030; *I*^2^ = 66.6%) and 90-day mortality (OR, 3.87; 95% CI, 2.64–5.68; *p* = 0.287; *I*^2^ = 19.3%) in patients with AC.

The similar rate of successful recanalization and symptomatic ICH in patients with AC and WC supports the safety of EVT in patients with AC. However, our study found that there was a significant difference in 90-day favorable outcome and mortality after EVT in patients with and without active cancer. The presence of analytical abnormalities or hemostatic abnormalities is common in cancer patients, and it may be a major cause for the poorer prognosis of these patients. The level of platelet in blood of cancer patients often decreases, which may result in the tendency of bleeding and a higher rate of complications. What is more, the growth of cancer consumes a large amount of body's energy and may bring to malnutrition, thus giving rise to the poor prognosis. In addition, EVT will inevitably lead to the injury of vessels and plaque and trigger the inner repairing mechanism. Accumulation of platelet is a reaction of inflammation, which is a stimulation to malignant cancer and is likely to worsen the post-surgery prognosis. It should be noticed that, according to recent articles, under certain circumstances, thrombosis is a physiological process that constitutes an intrinsic effector mechanism of innate immunity, whereas the rapid update of cancer cells will result in blood internal environment disorder and disable the defense system by thrombosis (22).

Intravenous thrombolysis (IVT), given to many patients in our meta-analysis, may be associated with higher risks in patients with AC. However, we did not observe a difference in hemorrhage rates and other studies have reported that IVT using alteplase is safe and effective for patients with AC (23, 24). The condition of cancer itself may also contribute to a higher rate of mortality. Stroke symptoms and related disability may influence decisions regarding cancer treatment, such as tumor resection and chemotherapy (5). Therefore, the death of patients in the AC group may be related to cancer and tumor progression. It is necessary to carry out more research on the stage, grade, and type of tumor to further analyze the mortality due to cancer.

Our study has several limitations. First, we could not evaluate the effects of cancer-related factors, including the cancer staging, brain metastasis, treatment status, and life expectancy due to insufficient data. Second, selection bias is inevitable in our study selection process. Furthermore, several included studies had small sample sizes, and there were inconsistent outcome measures. All of the included studies are retrospective, and only one was multicenter. More data are needed to confirm our conclusion.

## Conclusion

AC is likely to influence patient outcomes after EVT and may hold a higher risk of mortality. While there were similar rates of reperfusion and hemorrhage, further high-quality studies are warranted to better understand long-term outcomes.

## Data availability statement

The original contributions presented in the study are included in the article/Supplementary material, further inquiries can be directed to the corresponding author.

## Author contributions

LD contributed to the initial idea for this study. CS, QT, and XW developed and revised the search strategy. LD and HZ finished the study design. BY was consulted about the clinical issues. LD, ZF, and HZ contributed to the original draft. LD, ZF, HZ, CS, QT, AD, RR, ZS, XG, XW, and BY were responsible for the revision of the draft. All authors approved the final work before submission.

## Conflict of interest

The authors declare that the research was conducted in the absence of any commercial or financial relationships that could be construed as a potential conflict of interest.

## Publisher's note

All claims expressed in this article are solely those of the authors and do not necessarily represent those of their affiliated organizations, or those of the publisher, the editors and the reviewers. Any product that may be evaluated in this article, or claim that may be made by its manufacturer, is not guaranteed or endorsed by the publisher.
