# Supplementary material for: Outcomes after endovascular thrombectomy for acute ischemic stroke patients with active cancer: A systematic review and meta-analysis
Source: Front Neurol. 2022 Oct 20;13:992825. doi: 10.3389/fneur.2022.992825 (PMC9631814; doi:10.3389/fneur.2022.992825)
Supplement: Supplementary file 1 [file Data_Sheet_1.DOCX]

| **Ovid MEDLINE(R) and Epub Ahead of Print, In-Process, In-Data-Review & Other Non-Indexed Citations, Daily and Versions(R)** | | |
| --- | --- | --- |
| **序号** | **命中文献数** | **检索表达式** |
| 1 | 278070 | cerebrovascular disorders/ or basal ganglia cerebrovascular disease/ or exp brain ischemia/ or exp brain infarction/ or hypoxia-ischemia, brain/ or carotid artery diseases/ or carotid artery thrombosis/ or intracranial arterial diseases/ or cerebral arterial diseases/ or infarction, anterior cerebral artery/ or infarction, middle cerebral artery/ or infarction, posterior cerebral artery/ or exp "intracranial embolism and thrombosis"/ or stroke/ or exp ischemic stroke/ |
| 2 | 284476 | (stroke$ or apoplex$ or CVA).tw. |
| 3 | 143247 | ((brain or encephalic or cerebr$ or cerebell$ or vertebrobasil$ or hemispher$ or intracran$ or intracerebral or infratentorial or supratentorial or middle cerebr$ or MCA$ or anterior circulation or posterior circulation or basilar artery or vertebral artery or vertebrobasilar or space occupying or basal ganglia) adj5 (isch?emi$ or infarct$ or thrombo$ or emboli$ or occlus$ or hypoxi$ or stenos$ or narrow or constrit$ or strict$ or harden$ or insufficienc$ or arteriosclerosis or atherosclero$ or obstruct$ or block$)).tw. |
| 4 | 468206 | 1 or 2 or 3 |
| 5 | 3609717 | exp neoplasm/ or exp carcinoma/ or exp tumor/ or exp hematologic malignancy/ or exp leukemia/ or exp lymphoma/ or exp neoplasm metastasis/ |
| 6 | 4117899 | (cancer$ or neoplas$ or tumo$ or carcinom$ or malignan$ or oncolog$ or hodgkin$ or nonhodgkin$ or non-hodgkin$ or adenocarcinoma$ or adenoma$ or leuk?emia$1 or metasta$ or lymphoma$ or sarcom$ or melanoma$ or myeloma$ or epithelioma$ or glio$ or sarcoma$ or SCLC or NSCLC).tw. |
| 7 | 4861835 | 5 or 6 |
| 8 | 36515 | 4 and 7 |
| 9 | 223041 | endovascular procedures/ or catheterization/ or angioplasty/ or exp Angioplasty, Balloon/ or atherectomy/ or vascular surgical procedures/ or exp thrombectomy/ or mechanical thrombolysis/ or exp embolectomy/ or balloon embolectomy/ or exp stents/ |
| 10 | 457159 | (angioplast$ or stent$ or pta or revasculari?ation or recanali?ation or catheter$ or dilatation or thromboaspirat$ or thrombo-aspirat$ or thrombecto$ or embolecto$ or atherect$).tw. |
| 11 | 9122 | ((clot or thrombus or thrombi or embol$) adj5 (aspirat$ or remov$ or retriev$ or fragment$ or retract$ or extract$ or obliterat$ or dispers$ or obliterat$ or disrupt$ or disintegrate$)).tw. |
| 12 | 4543 | ((mechanical or pharmacomechanical or endovascular or neurovascular) adj5 (thrombolys$ or reperfusion or fragmentation or aspiration or recanali?ation or clot lys$)).tw. |
| 13 | 2080 | ((retrieval or extraction) adj5 device$).tw. |
| 14 | 233 | ((merci or concentric or penumbra or solitaire or trevo or tigertriever) adj5 retriever).tw. |
| 15 | 369 | (endovascular snare$ or microsnare or angiojet).tw. |
| 16 | 551689 | 9 or 10 or 11 or 12 or 13 or 14 or 15 |
| 17 | 2058 | 8 and 16 |
| 18 | 4913023 | (animals not humans).sh. |
| 19 | 1996 | 17 not 18 |
| 20 | 1712 | limit 19 to english language |

| **Cochrane Central Register of Controlled Trials (CENTRAL)** | | |
| --- | --- | --- |
| ID | Search | Hits |
| #1 | MeSH descriptor: [Cerebrovascular Disorders] this term only | 1456 |
| #2 | MeSH descriptor: [Basal Ganglia Cerebrovascular Disease] this term only | 11 |
| #3 | MeSH descriptor: [Brain Ischemia] explode all trees | 3886 |
| #4 | MeSH descriptor: [Brain Infarction] explode all trees | 1387 |
| #5 | MeSH descriptor: [Hypoxia-Ischemia, Brain] this term only | 241 |
| #6 | MeSH descriptor: [Carotid Artery Diseases] this term only | 499 |
| #7 | MeSH descriptor: [Carotid Artery Thrombosis] this term only | 20 |
| #8 | MeSH descriptor: [Intracranial Arterial Diseases] this term only | 12 |
| #9 | MeSH descriptor: [Cerebral Arterial Diseases] this term only | 27 |
| #10 | MeSH descriptor: [Infarction, Anterior Cerebral Artery] this term only | 7 |
| #11 | MeSH descriptor: [Infarction, Middle Cerebral Artery] this term only | 145 |
| #12 | MeSH descriptor: [Infarction, Posterior Cerebral Artery] this term only | 4 |
| #13 | MeSH descriptor: [Intracranial Embolism and Thrombosis] explode all trees | 329 |
| #14 | MeSH descriptor: [Stroke] this term only | 9962 |
| #15 | MeSH descriptor: [Ischemic Stroke] explode all trees | 181 |
| #16 | (stroke* or apoplex* or CVA):ti,ab,kw | 61840 |
| #17 | ((brain or encephalic or cerebr* or cerebell* or vertebrobasil* or hemispher* or intracran* or intracerebral or infratentorial or supratentorial or middle cerebr* or MCA* or anterior circulation or posterior circulation or basilar artery or vertebral artery or vertebrobasilar or space occupying or basal ganglia) near/5 (isch?emi* or infarct* or thrombo* or emboli* or occlus* or hypoxi* or stenos* or narrow or constrit* or strict* or harden* or insufficienc* or arteriosclerosis or atherosclero* or obstruct* or block*)):ti,ab,kw | 34031 |
| #18 | #1 or #2 or #3 or #4 or #5 or #6 or #7 or #8 or #9 or #10 or #11 or #12 or #13 or #14 or #15 or #16 or #17 | 84520 |
| #19 | MeSH descriptor: [Neoplasms] explode all trees | 85747 |
| #20 | MeSH descriptor: [Carcinoma] explode all trees | 14432 |
| #21 | MeSH descriptor: [Neoplasms] explode all trees | 85747 |
| #22 | MeSH descriptor: [Hematologic Neoplasms] explode all trees | 621 |
| #23 | MeSH descriptor: [Leukemia] explode all trees | 4911 |
| #24 | MeSH descriptor: [Lymphoma] explode all trees | 3403 |
| #25 | MeSH descriptor: [Neoplasm Metastasis] explode all trees | 5389 |
| #26 | (cancer* or neoplas* or tumo* or carcinom* or malignan* or oncolog* or hodgkin* or nonhodgkin* or non-hodgkin* or adenocarcinoma* or adenoma* or leuk?emia* or metasta* or lymphoma* or sarcom* or melanoma* or myeloma* or epithelioma* or glio* or sarcoma* or SCLC orNSCLC):ti,ab,kw | 257469 |
| #27 | #19 or #20 or #21 or #22 or #23 or #24 or #25 or #26 | 261353 |
| #28 | #18 and #27 | 4262 |
| #29 | MeSH descriptor: [Endovascular Procedures] this term only | 496 |
| #30 | MeSH descriptor: [Catheterization] this term only | 1652 |
| #31 | MeSH descriptor: [Angioplasty] explode all trees | 4502 |
| #32 | MeSH descriptor: [Angioplasty, Balloon] explode all trees | 4156 |
| #33 | MeSH descriptor: [Atherectomy] this term only | 26 |
| #34 | MeSH descriptor: [Vascular Surgical Procedures] this term only | 674 |
| #35 | MeSH descriptor: [Thrombectomy] explode all trees | 339 |
| #36 | MeSH descriptor: [Mechanical Thrombolysis] explode all trees | 42 |
| #37 | MeSH descriptor: [Embolectomy] explode all trees | 11 |
| #38 | MeSH descriptor: [Stents] explode all trees | 4451 |
| #39 | (angioplast* or stent* or pta or revasculari* or recanali* or catheter* or dilatation or thromboaspirat* or thrombo-aspirat* or thrombecto* or embolecto* or atherect*):ti,ab,kw | 66935 |
| #40 | ((clot or thrombus or thrombi or embol*) near/5 (aspirat* or remov* or retriev* or fragment* or retract* or extract* or obliterat* or dispers* or obliterat* or disrupt* or disintegrate*)):ti,ab,kw | 931 |
| #41 | ((mechanical or pharmacomechanical or endovascular or neurovascular) near/5 (thrombolys* or reperfusion or fragmentation or aspiration or recanali* or clot lys*)):ti,ab,kw | 767 |
| #42 | ((retrieval or extraction) near/5 device*):ti,ab,kw | 154 |
| #43 | ((merci or concentric or penumbra or solitaire or trevo or tigertriever) near/5 retriever):ti,ab,kw | 79 |
| #44 | (endovascular snare* or microsnare or angiojet):ti,ab,kw | 33 |
| #45 | #29 or #30 or #31 or #32 or #33 or #34 or #35 or #36 or #37 or #38 or #39 or #40 or #41 or #42 or #43 or #44 | 68176 |
| #46 | #28 and #45 in Trials | 583 |

| **EMBASE** | | |
| --- | --- | --- |
| No. | Query | Results |
| #18 | #8 AND #16 AND [english]/lim AND [humans]/lim | 4421 |
| #17 | #8 AND #16 | 5140 |
| #16 | #9 OR #10 OR #11 OR #12 OR #13 OR #14 OR #15 | 801400 |
| #15 | 'endovascular snare*':ab,ti OR microsnare:ab,ti OR angiojet:ab,ti | 656 |
| #14 | ((merci OR concentric OR penumbra OR solitaire OR trevo OR tigertriever) NEAR/5 retriever):ab,ti | 462 |
| #13 | ((retrieval OR extraction) NEAR/5 device*):ab,ti | 3100 |
| #12 | ((mechanical OR pharmacomechanical OR endovascular OR neurovascular) NEAR/5 (thrombolys* OR reperfusion OR fragmentation OR aspiration OR recanali* OR 'clot lys*')):ab,ti | 7865 |
| #11 | ((clot OR thrombus OR thrombi OR embol*) NEAR/5 (aspirat* OR remov* OR retriev* OR fragment* OR retract* OR extract* OR obliterat* OR dispers* OR obliterat* OR disrupt* OR disintegrate*)):ab,ti | 14521 |
| #10 | angioplast*:ab,ti OR stent*:ab,ti OR pta:ab,ti OR revasculari$ation:ab,ti OR recanali$ation:ab,ti OR catheter*:ab,ti OR dilatation:ab,ti OR thromboaspirat*:ab,ti OR 'thrombo aspirat*':ab,ti OR thrombecto*:ab,ti OR embolecto*:ab,ti OR atherect*:ab,ti | 706950 |
| #9 | 'endovascular surgery'/de OR 'angioplasty'/de OR 'percutaneous transluminal angioplasty'/de OR 'catheterization'/de OR 'catheter ablation'/de OR 'balloon dilatation'/de OR 'atherectomy'/exp OR 'stent'/de OR 'thrombectomy'/de OR 'percutaneous thrombectomy'/exp OR 'embolectomy'/exp OR 'bare metal stenting'/de | 286258 |
| #8 | #4 AND #7 | 81765 |
| #7 | #5 OR #6 | 6469925 |
| #6 | cancer*:ab,ti OR neoplas*:ab,ti OR tumo*:ab,ti OR carcinom*:ab,ti OR malignan*:ab,ti OR oncolog*:ab,ti OR hodgkin*:ab,ti OR nonhodgkin*:ab,ti OR 'non hodgkin*':ab,ti OR adenocarcinoma*:ab,ti OR adenoma*:ab,ti OR leuk$emia*:ab,ti OR metasta*:ab,ti OR lymphoma*:ab,ti OR sarcom*:ab,ti OR melanoma*:ab,ti OR myeloma*:ab,ti OR epithelioma*:ab,ti OR glio*:ab,ti OR sarcoma*:ab,ti OR sclc:ab,ti OR nsclc:ab,ti | 5616783 |
| #5 | 'malignant neoplasm'/exp OR 'metastasis'/exp OR 'neoplasms subdivided by anatomical site'/exp | 5076404 |
| #4 | #1 OR #2 OR #3 | 810835 |
| #3 | ((brain OR encephalic OR cerebr* OR cerebell* OR vertebrobasil* OR hemispher* OR intracran* OR intracerebral OR infratentorial OR supratentorial OR 'middle cerebr*' OR mca* OR 'anterior circulation' OR 'posterior circulation' OR 'basilar artery' OR 'vertebral artery' OR vertebrobasilar OR 'space occupying' OR 'basal ganglia') NEAR/5 (isch$emi* OR infarct* OR thrombo* OR emboli* OR occlus* OR hypoxi* OR stenos* OR narrow OR constrit* OR strict* OR harden* OR insufficienc* OR arteriosclerosis OR atherosclero* OR obstruct* OR block*)):ab,ti | 205922 |
| #2 | stroke*:ab,ti OR apoplex*:ab,ti OR cva:ab,ti | 454354 |
| #1 | 'cerebrovascular disease'/de OR 'cerebral artery disease'/de OR 'cerebrovascular accident'/de OR 'ischemic stroke'/exp OR 'cardioembolic stroke'/de OR 'lacunar stroke'/de OR 'vertebrobasilar insufficiency'/de OR 'carotid artery disease'/de OR 'carotid artery obstruction'/exp OR 'brain infarction'/exp OR 'brain ischemia'/exp OR 'occlusive cerebrovascular disease'/exp | 650336 |
